# Isoliquiritigenin Induces Autophagy and Inhibits Ovarian Cancer Cell Growth

**DOI:** 10.3390/ijms18102025

**Published:** 2017-09-21

**Authors:** Hsin-Yuan Chen, Tsui-Chin Huang, Tzong-Ming Shieh, Chi-Hao Wu, Li-Chun Lin, Shih-Min Hsia

**Affiliations:** 1 School of Nutrition and Health Sciences, College of Nutrition, Taipei Medical University, Taipei 11031, Taiwan; hsin246@gmail.com; 2 PhD Program for Cancer Biology and Drug Discovery, College of Medical Science and Technology, Taipei Medical University and Academia Sinica, Taipei 11031, Taiwan; tsuichin@gmail.com (T.-C.H.); lisa81318@gmail.com (L.-C.L.); 3 Department of Dental Hygiene, College of Health Care, China Medical University, Taichung 40402, Taiwan; tmshieh@mail.cmu.edu.tw; 4 Department of Human Development and Family Studies, National Taiwan Normal University, Taipei 106, Taiwan; chwu@ntnu.edu.tw; 5 Graduate Institute of Metabolism and Obesity Sciences, College of Nutrition, Taipei Medical University, Taipei 11031, Taiwan

**Keywords:** apoptosis, autophagy, isoliquiritigenin, ovarian cancer

## Abstract

Ovarian cancer is one of the commonest gynecologic malignancies, which has a poor prognosis for patients at the advanced stage. Isoliquiritigenin (ISL), an active flavonoid component of the licorice plant, previously demonstrated antioxidant, anti-inflammatory, and tumor suppressive effects. In this study, we investigated the antitumor effect of ISL on human ovarian cancer in vitro using the human ovarian cancer cell lines, OVCAR5 and ES-2, as model systems. Our results show that ISL significantly inhibited the viability of cancer cells in a concentration- and time-dependent manner. Flow cytometry analysis indicated that ISL induced G2/M phase arrest. Furthermore, the expression of cleaved PARP, cleaved caspase-3, Bax/Bcl-2 ratio, LC3B-II, and Beclin-1 levels were increased in western blot analysis. To clarify the role of autophagy and apoptosis in the effect of ISL, we used the autophagy inhibitor—3-methyladenine (3-MA) to attenuate the punctate fluorescence staining pattern of the p62/sequestosome 1 (SQSTM1, red fluorescence) and LC3 (green fluorescence) proteins after ISL treatment, and 3-MA inhibited the cytotoxicity of ISL. These findings provide new information about the link between ISL-induced autophagy and apoptosis and suggest that ISL is a candidate agent for the treatment of human ovarian cancer.

## 1. Introduction

Ovarian cancer is the fifth leading cause of death from all gynecological malignancies in developing countries, and it usually occurs in women after menopause [1]. In Taiwan, the incidence rate of ovarian cancer was 271.7 per 100,000 women per year based on cases reported in 1990–2013 in the 55–59 year age group [2]. Unfortunately, patients with ovarian cancer are usually diagnosed at the advanced stage because of the lack of obvious initial symptoms [3]. Therefore, oophorectomy is the first treatment choice for ovarian cancer. The currently available therapeutic options not only tumor debulking surgery, but also chemotherapy. However, drug resistance lead to chemotherapy failure and became the main reason for poor prognosis patient [4]. Therefore, adjuvant chemotherapy is required to prevent tumor relapse and metastasis. Isoliquiritigenin (ISL), a natural flavonoid isolated from the root of the licorice plant (*Glycyrrhiza uralensis*), has a chalcone structure (4, 20, 40-trihydroxychalcone) [5]. Recently, ISL has been increasingly recognized to possess cancer-preventive and -suppressive activities such as anti-inflammatory [6], antioxidant [7], anti-platelet aggregation [8], atheroprotective [9], and estrogenic effects [10]. ISL exerts its antitumor effects by altering multiple molecular targets and regulating apoptotic and autophagic activities [11,12]. More importantly, ISL has very little cytotoxic effects on normal tissues in vitro and in vivo at effective anticancer doses [13], indicating its potential value in cancer treatments when administered appropriately.

Autophagy is an important type II programmed cell death (PCD) involving the engulfment and degradation of abnormal cellular organelles or proteins, which are recycled to maintain cellular metabolism, viability, and homeostasis in living cells [14,15,16,17]. During the initiation of autophagic process, Beclin-1 can promoting the nucleation of the autophagosomal membrane, and then, entering the process of the elongation of the phagophore, ubiquitin-like conjugation of phosphatidylethanolamine (PE) to LC3 converts it to LC3-II, which is recruited to the autophagosomal membrane and has been widely used as a protein marker to indicate the occurrence of autophagy [18]. The two most common markers associated with autophagy are LC3B and p62/sequestosome 1 (SQSTM1). p62/SQSTM1 binds to LC3B through the LC3 interacting region (LIR) domain and is then degraded during the autophagic process. Therefore, the conversion of LC3BI to LC3BII and clearance of p62 are considered hallmarks of the autophagic flux. Autophagy is well identified to act as a tumor suppressor or tumor promoter in ovarian carcinoma [19]. Under basal cellular conditions, autophagy acts as a tumor suppressor and maintains the cellular turnover of proteins and organelles by lysosomal degradation [20]. In contrast, under nutrient-deprived stress conditions such as oxidative stress [20], autophagy promotes cellular adaptation and tumor survival by supplying macromolecules. Extensive autophagic activity eventually leads to cell death and, therefore, the potential of autophagy to act as a putative anticancer modality including the development of chemical inhibitors of autophagy as well as genetic silencing of key autophagy proteins have been investigated. Inhibiting autophagic activity in cells may switch their death signal responses from autophagic-type II programmed cell death to apoptotic type I cell death [21]. The aim of this study was to examine the effect of ISL on cell proliferation and whether inhibition of autophagy sensitized ovarian cancer cell lines to ISL in vitro.

## 2. Results

### 2.1. Inhibition of Cell Growth and Cell Cycle Progression at G2/M Phase in Ovarian Cancer Cells by Isoliquiritigenin

**Figure 1:**
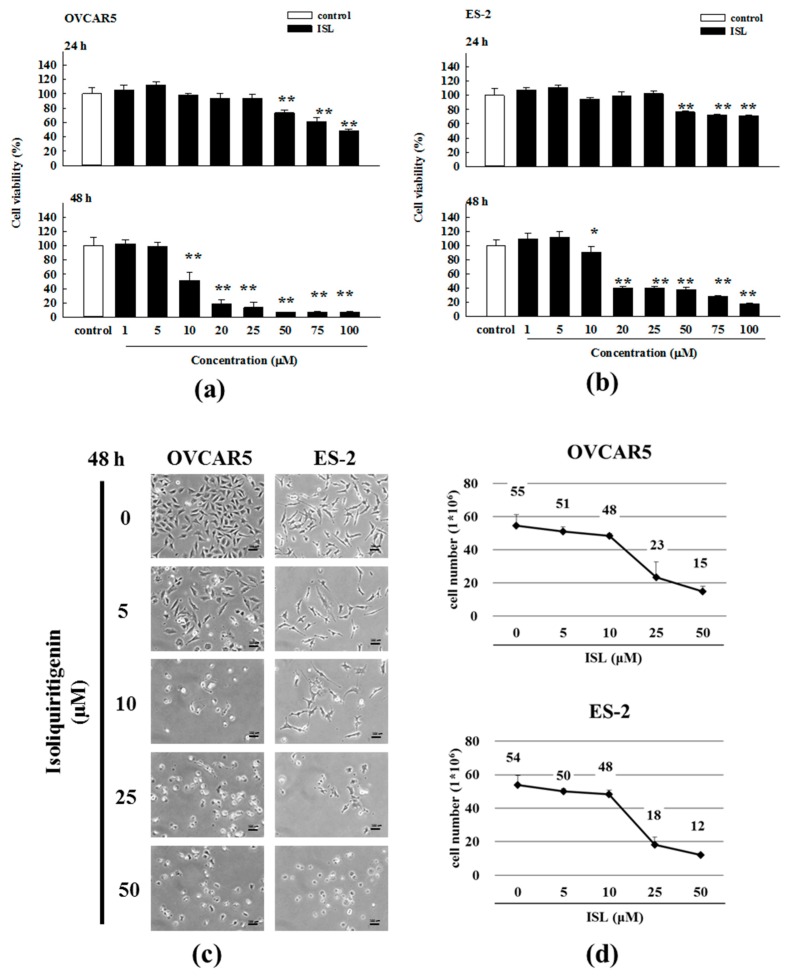
Cytotoxicity of isoliquiritigenin on ovarian cancer cells. (**a**) OVCAR5 and (**b**) ES-2 cells were exposed to control (dimethyl sulfoxide, DMSO) and the indicated concentration of ISL from 1 to 100 μM follow by 24–48 h incubation. Cell proliferation was measured by MTS assay; (**c**) The morphology of OVCAR5 and ES-2 cells after treated with control (DMSO) and 5, 10, 25, and 50 μΜ of ISL in media containing 1% FBS for 48 h. Bars equal to 100 μm; (**d**) Number of viable cells after treated with control (DMSO) and 5, 10, 25, and 50 μΜ of ISL in media containing 1% fetal bovine serum (FBS) for 48 h. Cell viability was determined by trypan blue exclusion test. The results are expressed as means ± SD of three independent experiments. * *p* < 0.05 and ** *p* < 0.001 compared with control.

**Figure 2:**
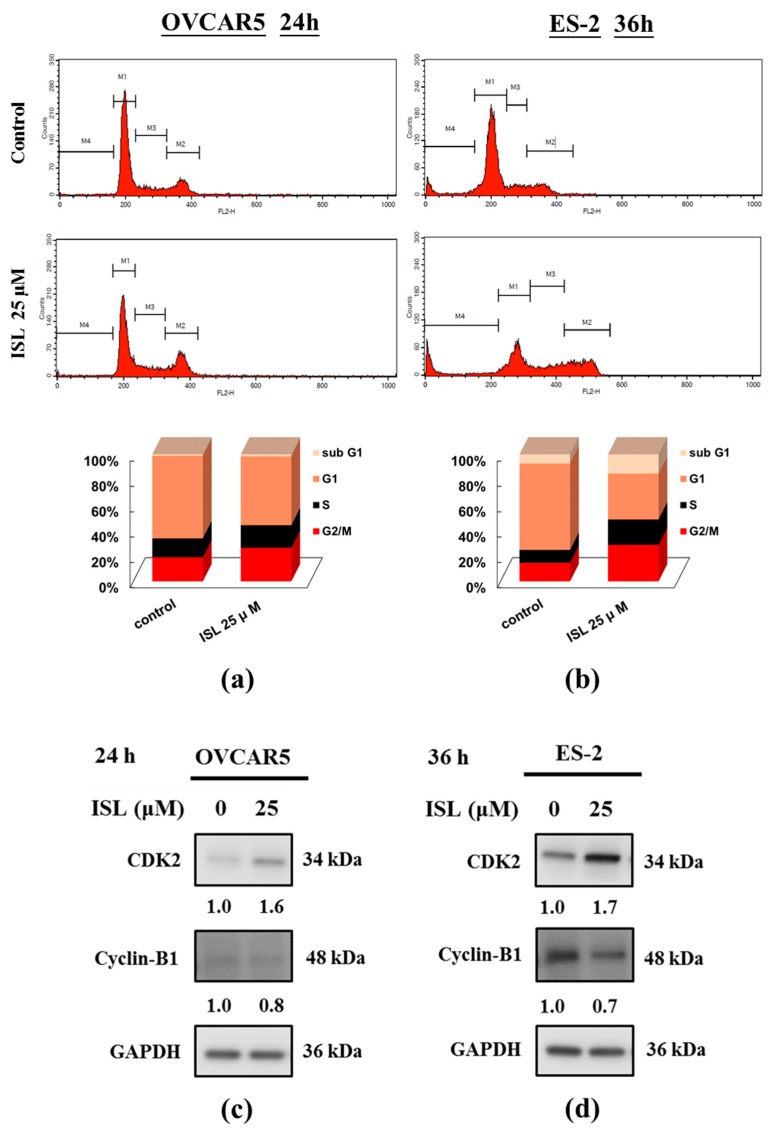
ISL induces G2/M cell cycle arrest in ovarian cancer cells. Cells were plated in 100 mm diameter dishes at 1 × 10^6^ cells in medium with 10% FBS until attach the plate bottom and treated with ISL 25 μM for 24 or 36 h. (**a**,**b**) The cells were stained with propidium iodide (PI), and the cell cycle distribution was analyzed by flow cytometry. The vertical axis represents the number of cells and the horizontal axis represents the intensity of PI staining. The cell cycle distribution was shown in bar graph. The vertical numbers represents the cell population percentage in cell cycle sub G1, G1, S and G2/M phase, the horizontal number represents the dose of ISL; (**c**,**d**) Cell lysates were separated by sodium dodecyl sulfate polyacrylamide gel electrophoresis (SDS-PAGE) and analyzed on western blots with the indicated antibodies. GAPDH was used as a loading control. The values of the band intensity represent the densitometric estimation of each band normalized by GAPDH.

To evaluate the cytotoxic effect of ISL, ovarian cancer cells (OVCAR5 and ES-2) were treated with ISL (0, 1, 5, 10, 20, 25, 50, 75, and 100 μM) for 24 and 48 h, and cell viability was measured using the 3-(4,5-dimethylthiazol-2-yl)-5-(3-carboxymethoxyphenyl)-2-(4-sulfophenyl)-2*H*-tetrazolium (MTS) assay. ISL decreased the growth of OVCAR5 and ES-2 cells in a concentration- and time-dependent manner (Figure 1a,b), and we also calculated that the half maximal inhibitory concentration (IC_50_) is 11 μM or 25 μM for ISL in OVCAR5 or ES-2 cells. In addition, we using the trypan blue exclusion test to examine the cell viability of OVCAR5 and ES-2, as Figure 1d show, ISL inhibited the growth of ovarian cancer cells and rounded floating cells were observed (Figure 1c,d). To further investigate whether the cytotoxicity of ISL is associated with cell cycle arrest, we examined its effects on cell cycle progression and results showed that ISL 25 μM arrested the cell cycle at the G2/M checkpoint phase (Figure 2a,b) and caused cyclin-dependent kinase 2 (CDK2) protein increased while the cyclin B1 decreased in OVCAR5 and ES-2 cells (Figure 2c,d).

### 2.2. Effects of ISL on Apoptosis- and Autophagy-Associated Protein Expression

**Figure 3:**
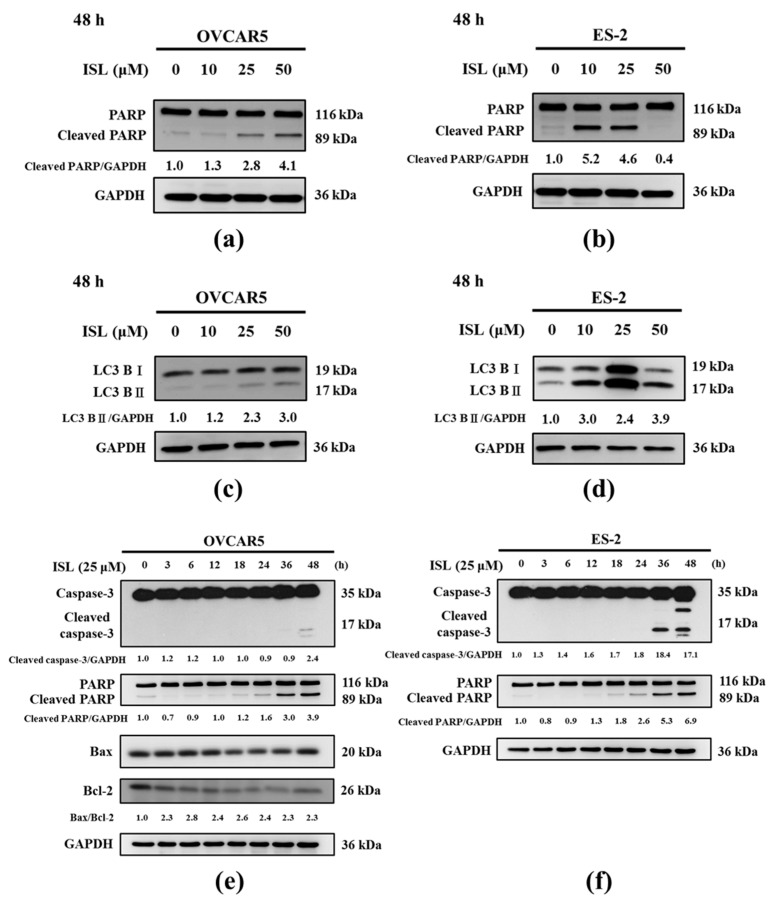

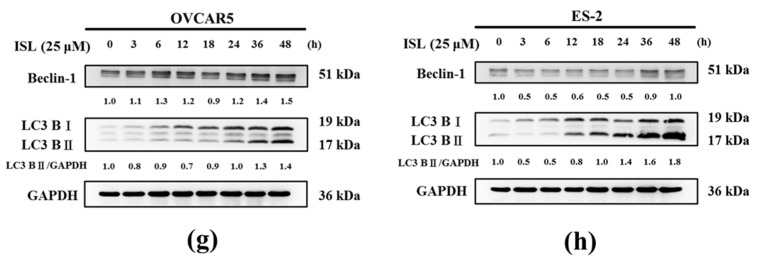
ISL induces the expression of autophagy and apoptosis-associated protein in ovarian cancer cells. OVCAR5 and ES-2 cells were treated with ISL (10, 25, 50 μM) for 48 h (**a**–**d**) and treated with ISL 25 μM for 3, 6, 12, 18, 24, 36, and 48 h (**e**–**h**). Cell lysates were separated by SDS-PAGE and analyzed on western blots with the indicated antibodies. GAPDH was used as a loading control. The values of the band intensity are expressed as the ratio (cleaved PARP or LC3B-II or cleaved caspase-3 or Bax/Bcl-2 or Beclin-1:GAPDH) relative to control.

Then, we investigated whether ISL induced apoptosis and autophagy of ovarian cancer cells. After treatment with ISL (10, 25, and 50 μM) for 48 h, the protein expression levels of cleaved poly-ADP-ribose polymerase (PARP) and LC3B-II were increased in OVCAR5 and ES-2 cells, especially at 25 μM (Figure 3a–d). Based on the above results, we selected ISL 25 μM as the concentration for the subsequent experiments. We found the apoptosis-associated protein (cleaved caspase-3, cleaved PARP, and Bax/Bcl-2 ratio) levels were increased in OVCAR5 and ES-2 cells after ISL 25 μM treatment (Figure 3e,f). In addition, the autophagy-associated marker, LC3B-II and Beclin-1, were used in our study. As shown in Figure 3g,h, ISL 25 μM treatment also significantly increased the levels of LC3B-II and Beclin-1 in OVCAR5 and ES-2 cells.

### 2.3. ISL Triggers Autophagy or Apoptotic Cell Death of Ovarian Cancer Cells

**Figure 4:**
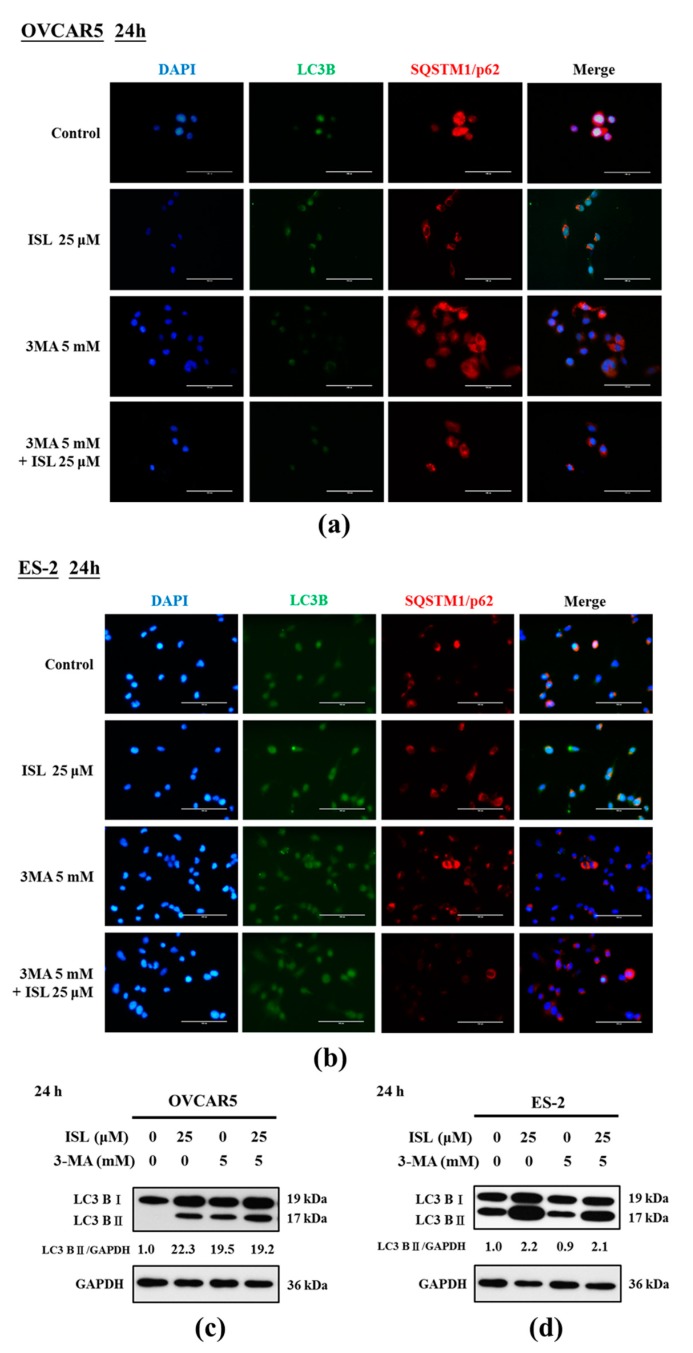
ISL triggered autophagy in ovarian cancer cells. OVCAR5 and ES-2 cells were treated with ISL 25 μM for 24 h with or without autophagy inhibitor (3-MA) pretreatment (5 mM, 4 h), and observed the co-localization of LC3 puncta (green) and p62/SQSTM1 puncta (red, demarcation for the lysosome) in cells, DAPI (4′,6-Diamidino-2-Phenylindole) was used for nucleus staining. Bars equal to 100 μm (**a**,**b**). OVCAR5 and ES-2 cell lysates were separated by SDS-PAGE and analyzed on western blot with autophagosome formation marker LC3B-I and LC3B-II. GAPDH was used as a loading control. The values of the band intensity are expressed as the ratio LC3B-II:GAPDH relative to control (**c**,**d**).

**Figure 5:**
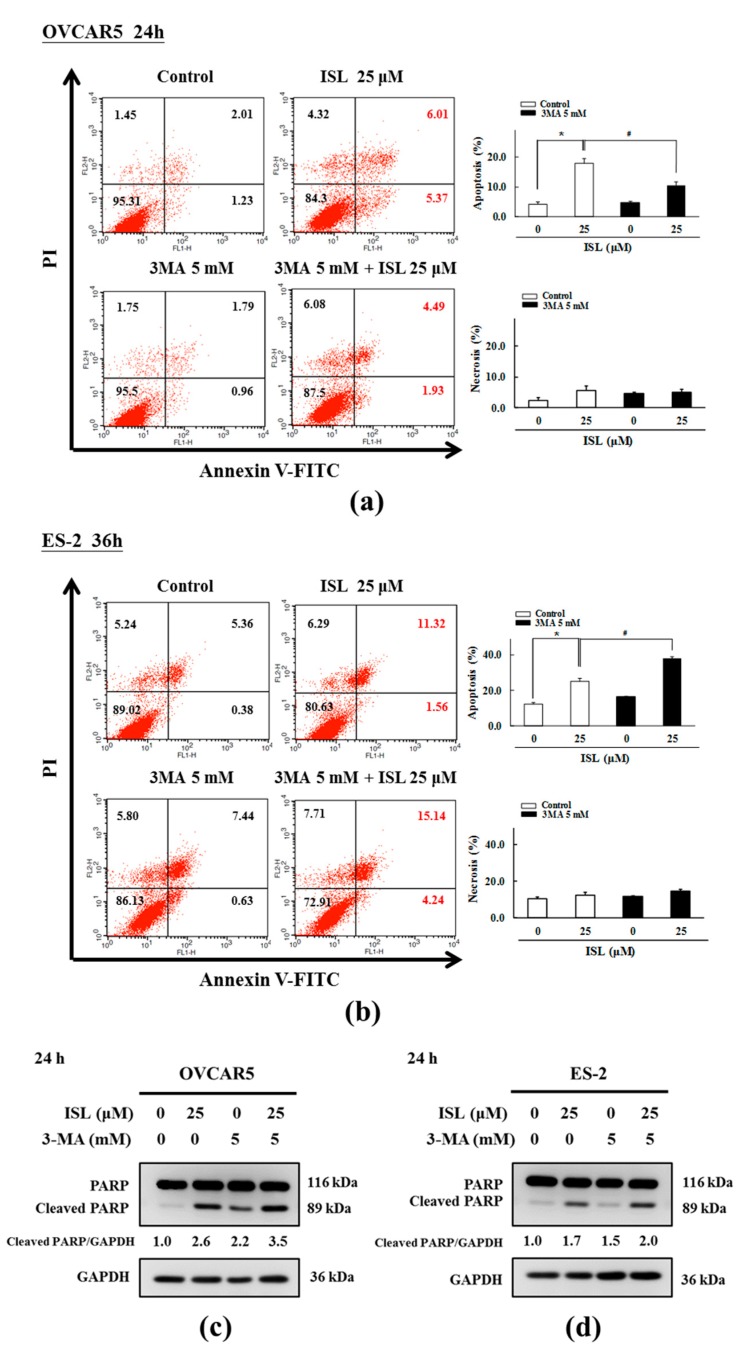
ISL induced apoptosis in ovarian cancer cells. OVCAR5 and ES-2 cells were treated with ISL 25 μM for 24 or 36 h with or without autophagy inhibitor (3-MA) pretreatment (5 mM, 4 h), cells were harvested and stained with Annexin V-FITC (fluorescein isothiocyanate) and PI (propidium iodide), and apoptotic cell death was analyzed using flow cytometry, relative proportions of both early and late apoptosis are indicated in right lower and right upper quadrant, respectively in each treatment group (**a**,**b**). OVCAR5 and ES-2 cell lysates were separated by SDS-PAGE and analyzed on western blots with the apoptosis markers total PARP and cleaved PARP. GAPDH was used as a loading control. The values of the band intensity are expressed as the ratio cleaved PARP:GAPDH relative to control (**c**,**d**). * *p* < 0.05 compared with control group; ^#^ *p* < 0.05 compared with ISL 25 μΜ group.

To clarify the effect of ISL-induced autophagy in OVCAR5 and ES-2 cells, we evaluated the effects of ISL on cell survival and apoptosis in cells pretreated with the autophagy inhibitor 3-methyladenine (3-MA). Immunocytochemistry staining showed that ISL 25 μM induced the expression of LC3 in OVCAR5 and ES-2 cells, which accommodated the development of numerous large autophagic vacuoles in the cytoplasm. However, the fluorescence intensity of LC3B was decreased, and p62/SQSTM1 protein (a marker of autophagic degradation) increased in ISL-treated OVCAR5 and ES-2 cells pretreated with 3-MA (5 mM, 4 h) (Figure 4a,b). Then, we assessed whether ISL induces the apoptosis of OVCAR5 and ES-2 cells using the Annexin V-fluorescein isothiocyanate (FITC) and propidium iodide (PI) double staining, which revealed the significant presence of Annexin V-FITC-positive cells after ISL treatment of OVCAR5 and ES-2 cells. However, the results differed between both cell lines; the number of Annexin V-FITC-positive OVCAR5 cells decreased while the positivity increased in ES-2 cells pretreated with 3-MA (Figure 5a,b). Consistent with the immunocytochemistry analysis, western blot analysis indicated that ISL 25 μM treatment further decreased conversion of LC3B-I to LC3B-II (Figure 4c,d) following 3-MA treatment, and obviously enhanced the protein levels of cleaved PARP in OVCAR5 and ES-2 cells (Figure 5c,d). Collectively, these results provide evidence that ISL-induced autophagy is tightly linked to apoptosis, and demonstrated that ISL triggered a protective autophagy in ES-2 cells. In contrast, ISL triggered autophagic cell death of OVCAR5 cells. Further investigations would be necessary to elucidate the relationship between autophagy and apoptosis after ISL treatment of OVCAR5 and ES-2 cells.

## 3. Discussion

To the best of our knowledge, this is the first study that directly demonstrates the beneficial effects of ISL in ovarian cancer. We provided two different pieces of evidence showing that the pharmacological inhibition of autophagy potentiated the ISL-induced autophagic death of OVCAR5 cells while, in contrast, autophagy played a protective role in ES-2 cells in vitro. ISL has pleiotropic functions in both in vivo and in vitro experimental models [22,23,24,25,26] including antioxidant, anti-inflammatory, and anticarcinogenic effects. In our previous study, we also found that ISL suppressed the growth of human endometrial cancer in vitro and in vivo [13] and inhibited the growth of human uterine sarcoma cells by increasing the chemosensitivity of doxorubicin [27]. Although its potential role in cancer therapy has been studied for decades, the effects of ISL in the prevention and treatment of ovarian cancer are still unclear. Similar to our previous study [13], the concentrations of ISL (1–100 μΜ) used in the experiment were based on a preliminary screening. ISL at concentrations up to 25 μΜ significantly suppressed ovarian cancer cell growth and reduced the survival rate by approximately 50%.

ISL reportedly inhibits the growth of different cancer cells by inducing cell cycle arrest such as G2/M phase arrest in oral squamous cell carcinoma cells [28], and G1 or G2/M phase arrest in prostate cancer cells [29]. Furthermore, our previous study also showed that ISL induced G1 or G2/M phase arrest in endometrial cancer cells [13]. In classical cell cycle model, each phase is driven by unique cyclin-dependent kinases (CDKs) bound to specific cyclins [30]. Previous studies have reported several characteristic changes in key cell cycle regulators such as CDC25c, CDK2, CyclinB1, etc following G2/M phase arrest [31]. CDK2 is subsequently activated by cyclin A during the late stages of DNA replication to drive the transition from S phase to mitosis, a period known as the G2 phase [30]. Entry into mitosis relies on an increase in the concentration of cyclin B1/CDK1 complexes by the rise of cyclin B1 during G2-phase and its accumulation during mitosis [32]. Cheng et al. [33] found that sulforaphane (SFN) arrested cell growth in the G2/M phase via down-regulation of Cyclin B1 gene expression, dissociation of the cyclin B1/CDC2 complex, and up-regulation of GADD45β proteins. Similar to previous results, we found that ISL induced G2/M phase arrest via up-regulation of CDK2 protein expression, while down-regulation of Cyclin B1 protein expression in OVCAR5 and ES-2 cells.

In addition to promoting cell cycle arrest, ISL is known to induce apoptosis of a variety of human cancer cells. Hirchaud et al. [34] found that ISL induces apoptosis by stimulating caspase activities and induced the cleavage of caspase-3 and its downstream substrate PARP in Ca Ski cells. Kim et al. [35] also demonstrated that S-ISL promoted apoptosis in Tca8113 cells via changing the levels of the Bcl-2 family and the ratio of Bax and Bcl-2. In addition, the alteration of intracellular Bax and Bcl-2 expression ratio can affect mitochondrial content release [36] and determine susceptibility to apoptosis [37]. In agreement with the previous studies, treatment with ISL 25 μΜ significantly induced apoptosis by upregulating the expression of cleaved-caspase-3, cleaved-PARP, and Bax/Bcl-2 ratio in OVCAR5 and ES-2 cells. These data suggest that apoptosis plays a key role in the ISL-induced cancer cell death. However, more studies are needed to clarify the role of the apoptotic factors in ovarian cancer cells.

According to a previous study, the expression of LC3 was dramatically higher in benign and borderline ovarian tumor samples than it was in malignant ovarian cancer samples. In addition, the expression of LC3 was higher in International Federation of Gynecology and Obstetrics (FIGO) stages I and II than it was in stages III and IV [38]. In our study, we also found that the LC3 expression increased after ISL treatment of OVCAR5 and ES-2 cells. Beclin-1 induces the formation of autophagosomes by activating phosphatidylinositol 3-kinase (PI3K) and, thereby, increasing autophagy. Higher levels of Beclin-1 were negatively associated with advanced FIGO stage and histological grade [38]. In our study, we also found that the expression of Beclin-1 was increased after ISL treatment of OVCAR5 and ES-2 cells. p62/SQSTM1 is an ubiquitin-binding scaffold protein that targets ubiquitinated protein aggregates for selective degradation by autophagy. In a previous study, a high expression level of cytoplasmic p62 in ovarian cancer cells was positively correlated with serous carcinoma, advanced stage, the presence of residual tumors, and a low overall survival rate [39]. In our study, we also found that the formation of punctate p62/SQSTM1 decreased after ISL treatment of OVCAR5 and ES-2 cells. In brief, Beclin-1 and LC3B-II are localized on the autophagosomal membrane accompanied with the downregulation of p62/SQSTM1 during autophagy [40].

Natural compound-induced autophagy may be pro-survival or pro-death in cancer therapy. Accumulating evidence shows that autophagy inhibition combined with anticancer agents can be a novel and effective therapy for treating various human malignancies [41]. 3-MA was the first specific autophagy inhibitor identified and has been widely used since 1982. Seglen et al. [42] isolated hepatocytes from starved rats to screen purine-related substances [42]. Subsequent studies confirmed that 3-MA, together with LY294002 and wortmannin, suppresses autophagy by inhibiting PI3K type III [43,44]. We speculated that the inhibition of autophagy enhances the cytotoxic effects of ISL by its survival mechanism under harsh conditions. As expected, combined treatment with ISL and 3-MA attenuated the expression of LC3B-II, but increased cleaved PARP levels in ES-2 cells and induced higher apoptotic cell death than ISL treatment did alone in vitro. These results are also similar to those of a previous study that found that the inhibition of autophagy in malignant glioma cell lines exposed to BAF-1 and arsenic trioxide led to cell death by apoptosis instead [45]. Furthermore, the inhibition of autophagy potentiated atorvastatin-induced apoptotic cell death in human bladder cancer cells in vitro [46]. The above results show that autophagy plays a protective role. Interestingly, different results were observed in the other ovarian cancer cells in which combined treatment with ISL and 3-MA produced lower apoptotic cell death than ISL treatment did alone in OVCAR5 cells. In our previous study, ISL also decreased the expression of LC3B-II and cleaved PARP following 3-MA treatment, and significantly decreased the viability of HEC-1A cells [13]. The above results showed autophagic cell death, indicating that autophagic cells were present in regions where cell death occurred. Thus, these research findings suggest that autophagy plays dual roles in cancer.

There are some limitations to the present study that are worth mentioning. First, we used only one chemical autophagy inhibitor to explore the role of autophagy in ISL-mediated apoptotic cell death. Autophagy inhibitors are classified as early- or late-stage drugs according to their mechanisms of action on the pathway inducing autophagy [47,48]. Early-stage inhibitors include 3-MA, wortmannin, and LY294002 while late-stage inhibitors include the antimalarial drugs chloroquine (CQ), hydroxychloroquine (HCQ), bafilomycin A1 (BAF-1), and monensin [49]. In this study, we only used 3-MA as an early stage inhibitor and CQ as a late-stage inhibitors (Figure S1). Thus, other interventions such as the chemical late stage inhibitors BAF-1 or genetic ablation using knockout and siRNA silencing of essential autophagy genes should be used to clarify the effect of autophagy in ovarian cancer cells. Secondly, the underlying mechanisms of ISL remain to be elucidated in detail and, therefore, further exploration of the molecular mechanisms and biological significance of ISL on autophagy induction is warranted.

## 4. Materials and Methods

### 4.1. Reagents and Antibodies

McCoy’s 5A medium, sodium bicarbonate, dimethyl sulfoxide (DMSO), DNAse-free RNAse A, PI and Triton X-100 were purchased from Sigma-Aldrich (St. Louis, MO, USA). Roswell Park Memorial Institute (RPMI) 1640 medium, antibiotic-antimycotic solution (100×) and 0.05% trypsin-ethylenediaminetetraacetic acid (EDTA (1×) were purchased from CAISSON Labs (Smithfield, UT, USA). Fetal bovine serum (FBS), trypan blue, bicinchoninic acid (BCA) protein assay kit, and electrochemiluminescence (ECL) immunoassay were purchased from Thermo Fisher Scientific (Logan, UT, USA). Bovine serum albumin (BSA) was purchased from BioShop (Burlington, ON, Canada). Protease and phosphatase inhibitor cocktail tablets were purchased from Roche. CellTiter 96^®^ AQueous One Solution cell proliferation assay (MTS assay) were purchased from Promega (Madison, WI, USA). Annexin V-FITC apoptosis detection kit I were purchased from Becton Dickinson (BD) Biosciences (San Jose, CA, USA). 3MA was purchased from Cayman Chemical Company (Ann Arbor, MI, USA). The following antibodies were used in this study: anti-LC3B, anti-Beclin-1, anti-PARP, anti-Bax, anti-AKT, anti-p-AKT (Ser473), mTOR, p-mTOR (Cell Signal Technology, Danvers, MA, USA), horseradish (HRP)-conjugated anti-glyceraldehyde 3-phosphate dehydrogenase (GAPDH, Proteintech, Rosemont, IL, USA), anti-Cyclin B1, anti-Bcl-2 (GeneTex, Irvine, CA, USA), anti-SQSTM1/p62, anti-CDK-2, and goat anti-rabbit/mouse antibody IgG (Abcam, Cambridge, MA, USA). The goat anti-rabbit/mouse antibody IgG Alexa Fluor^®^ 488/546 dye was from Thermo Fisher Scientific.

### 4.2. Preparation of ISL

ISL (C15H12O4, CAS number: 961-29-5), a yellow needle-shaped crystalline powder that is insoluble in water, was purchased from Cayman Chemical Company (purity < 98%). A stock solution of 100 mM was prepared in DMSO, aliquoted, and then stored at −20 °C until use. For all experiments, the final concentrations of the test compound were prepared by diluting the stock with cell culture medium. The control cells were treated with the vehicle solvent (0.1% DMSO).

### 4.3. Cell Culture

The human ovarian cancer cell lines (OVCAR5 and ES-2) were purchased from Food Industry Research and Development Institute (FIRDI) and Culture Collection and Research Center (CCRC, Taiwan, ROC). OVCAR5 cell lines were cultured in RPMI1640 and ES-2 cell line was cultured in McCoy’s 5A. Both cell lines were maintained in medium containing 10% FBS and 1% antibiotics (10,000 units/mL penicillin, 10,000 μg/mL streptomycin, 25 μg/mL amphotericin with 8.5 g/L NaCl) and incubated at 37 °C with 5% CO_2_.

### 4.4. Cell Viability Assay

The effect of ISL treatment on cell viability was examined using the MTS assay. Cells were seeded in 96-well plates (2 × 10^3^ cells/well), cultured for 24 h, and then treated with various concentrations of ISL in fresh medium containing 1% FBS. The reagent mixture (MTS/PMS) was added directly to each well for 24 and 48 h at a recommended ratio of 20:100 μL of reagent:culture medium. The absorbance was measured at 490 nm with a reference wavelength of >630 nm using an enzyme-linked immunosorbent assay (ELISA) reader (BioTek, Winooski, VT, USA).

### 4.5. Trypan Blue Exclusion Test

After the cells had adhered, the medium was replaced with fresh medium containing 1% FBS and various concentrations of ISL. After 48 h incubation, the cells were harvested from the culture dishes using culture medium with 0.05% trypsin-EDTA solution, collected by centrifugation for 5 min at 4 °C, and then evenly resuspended in 1 mL medium. Furthermore, the cells in 10 μL aliquots were counted using a hemocytometer using trypan blue staining to determine the number of surviving and dead cells. The cell counting method used involved sequentially counting the total number of living cells on four squares (top left and right, bottom left and right) of a counting disk (cell counter), and the total number of cells was determined using the derivation formula.

### 4.6. Cell Cycle Analysis

To assess the cell cycle progression, the cells were seeded into culture dishes (1 × 10^6^ cells), incubated for 24 h to allow exponential growth, and then they were treated with ISL at the indicated concentrations. All the cells were collected, slowly added to 9 mL 70% ethanol, and then they were stored at −20 °C for at least 2 h. Then, the cells were washed at least once with cold phosphate-buffered saline (PBS), resuspended in 300–500 µL PI/Triton X-100 staining solution (10 mL 0.1% (*v*/*v*) Triton X-100 in PBS containing 2 mg DNAse-free RNAse A and 0.40 mL of 500 µg/mL PI), and incubated for 30 min at 20 °C. The fluorescence was measured using a fluorescence-activated cell-sorting (FACS) Calibur flow cytometer (BD, San Jose, CA, USA) and the cell cycle distribution was analyzed using CellQuest and Modfit LT programs (BD).

### 4.7. Apoptosis Analysis

For the apoptosis analysis, a commercial Annexin V-FITC apoptosis detection kit was used. The cells were seeded in culture dishes (1 × 10^6^ cells), treated with ISL at the indicated concentrations, and then stained with Annexin V-FITC and PI by incubation for 15 min at room temperature protected from light. The apoptotic cells were analyzed using a FACS Calibur flow cytometer, and the results were analyzed using the CellQuest software program.

### 4.8. Western Blot Analysis

The ovarian cancer cell line lysates were prepared in ice-cold lysis buffer (50 mmol/L Tris (pH 8.0), 100 mmol/L sodium chloride (NaCl), 0.1% sodium dodecyl sulfate (SDS), 1% NP-40, 0.5 mM EDTA) containing the protease or phosphatase inhibitor cocktail or both. The proteins (20 μg) were boiled for 5 min, separated using 15 or 7.5% SDS-polyacrylamide gel electrophoresis (PAGE), and then they were transferred electrophoretically to Immobilon-P polyvinylidene fluoride (PVDF) membranes (0.22-µm) for 150–180 min at 280 mA and 250 V. Then, the membranes were washed thrice for 10 min each with Tris-buffered saline (TBS) plus Tween 20 (TBST) buffer, blocked with blocking buffer (5% BSA) for 1 h at room temperature, and incubated overnight with primary antibodies (1:1000 in blocking buffer) at 4 °C. The next day, the membranes were washed thrice for 10 min each time with TBST buffer, incubated for 1 h in blocking buffer with anti-rabbit/mouse IgG coupled to alkaline phosphatase (1:10,000), washed thrice for 10 min each time with TBST buffer, and then finally the bands were detected using ECL. The values shown were quantified, normalized to the internal control GAPDH, and then the densitometric estimation was performed using the ImageJ software.

### 4.9. Immunocytochemistry Analysis

Cells were cultured on sterile glass coverslips (1 × 10^4^ cells) and treated with ISL (25 μM for 24 h) followed by 3MA (5 mM for 4 h). After treatment, the cells were fixed with 4% paraformaldehyde solution at room temperature for 10 min, rinsed twice in 1× PBS, permeabilized using 0.1% Triton X-100/PBS for 5 min, and then blocked in blocking solution (5% (*w*/*v*) BSA in 1× TBST). The cells were incubated with the primary antibodies overnight at 4 °C, rinsed thrice in 1× PBS for 15 min, and then incubated with the secondary antibodies for 1 h at room temperature, followed by four rinses in 1× TBST for 20 min each. The cells were then treated with ProLong^®^ Gold antifade mountant (Thermo Fisher Scientific) and the fluorescent images were captured using an EVOS^®^ microscope (Thermo Fisher Scientific).

### 4.10. Statistical Analysis

The data were presented as the mean ± standard deviation (SD) and the differences between the means were analyzed using a one-way analysis of variance (ANOVA) using the statistical package for the social sciences (SPSS) program version 11.0 (SPSS, Chicago, IL, USA). The group means were compared using a one-way ANOVA and Duncan’s multiple-range test. For comparison of two groups, the Student’s *t*-test was used. The difference between two means was considered statistically significant when *p* < 0.05 and highly significant when *p* < 0.01.

## 5. Conclusions

The present study demonstrated that ISL induced cell cycle G2/M phase arrest and apoptosis of ovarian cancer cells. As expected, combined treatment with ISL and 3-MA attenuated the expression of LC3B-II, but produced higher apoptotic cell death than ISL treatment did alone in ES-2 cells, which indicated that autophagy played a protective role. Interestingly, the same treatment attenuated apoptotic death of OVCAR5 cells, which suggest that autophagic cell death occurred. These results indicate that autophagy plays dual roles in ovarian cancer and further studies should be performed to clarify the role of autophagy in ovarian cancer cells.

## Supplementary Materials

Supplementary materials can be found at www.mdpi.com/1422-0067/18/10/2025/s1.

## Abbreviations

BAF-1	Bafilomycin A1
CDK2	Cyclin-dependent kinase 2
CQ	Chloroquine
HCQ	Hydroxychloroquine
ISL	Isoliquiritigenin
LC3	Microtuble-associated protein light chain 3
PARP	Poly (ADP-ribose) polymerase
P62/SQSTM1	p62/Sequestosome 1
3-MA	3-Methyladenine
SDS-PAGE	Sodium dodecyl sulfate polyacrylamide gel electrophoresis
FBS	Fetal bovine serum
GAPDH	Glyceraldehyde 3-phosphate dehydrogenase
MTS	[3-(4,5-dimethylthiazol-2-yl)-5-(3-carboxymethoxyphenyl)-2-(4-sulfophenyl)-2H-tetrazolium
PMS	Phenazine methosulfate
