# Supplementary material for: Isoliquiritigenin Induces Autophagy and Inhibits Ovarian Cancer Cell Growth
Source: Int J Mol Sci. 2017 Sep 21;18(10):2025. doi: 10.3390/ijms18102025 (PMC5666707; doi:10.3390/ijms18102025)
Supplement: Supplementary file 1 [file ijms-18-02025-s001.pdf]

Supplementary

ISL triggers apoptotic cell death via Akt/mTOR pathway

To further investigate the mechanism of autophagy after ISL treatment, we used CQ, an inhibitor of autophagy, to determine the role of ISL-induced autophagy. ES-2 cells were treated with 50 and 100 μM CQ for 4 h before ISL treatment. As shown in Supplement Fig 1, the expression of p-Akt and p-mTOR were decreased in co-treatment with ISL and CQ group. However, the expression of LC3-II in ES-2 cells were remarkably up-regulated in response to co-treatment with ISL and CQ, suggesting that ISL induces autophagic flux in ovarian cancer cells. In addition, the expression of cleaved PARP was much more noticeable expressed in co-treatment with ISL and CQ group. Based on the above results, we suppose that ISL may trigger apoptotic cell death via Akt/mTOR pathway.

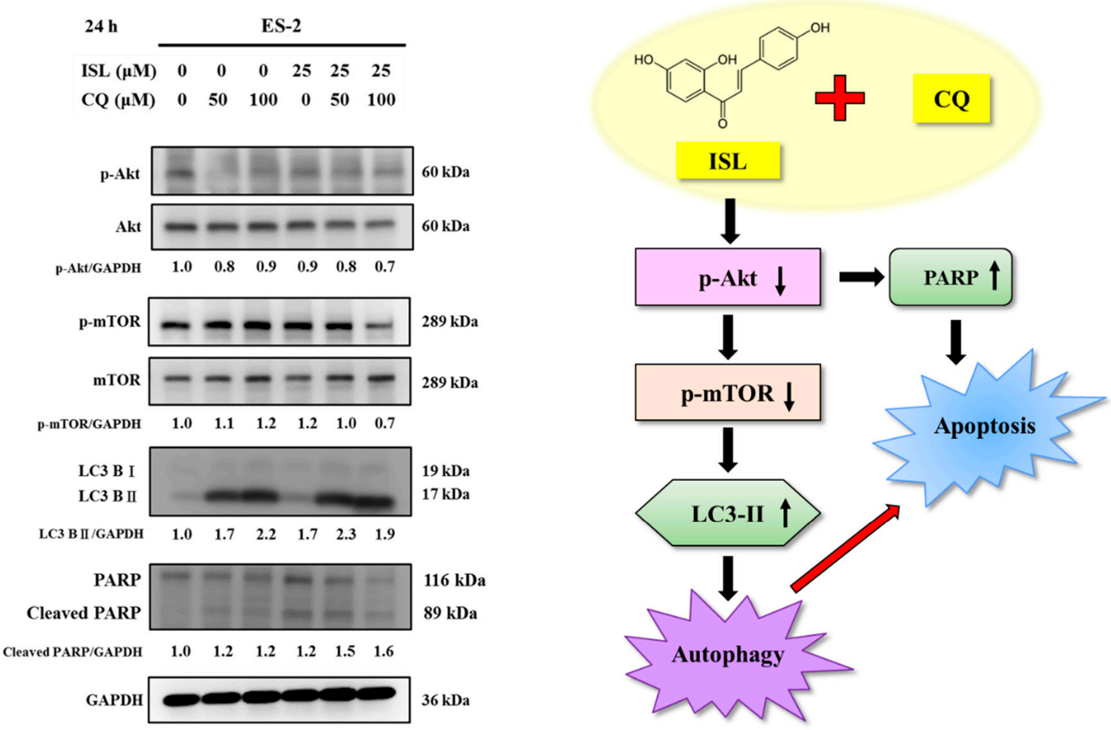

(a) (b)

Figure S1. Pharmacological inhibition of autophagy promotes ISL-induced cell death in ES-2 cells. ES-2 cells were treated with ISL 25 μM for 24 h with or without autophagy inhibitor (Chloroquine, CQ) pretreatment (50, 100 μM, 4 h), cell lysates were separated by SDS-PAGE and analyzed on western blot with the indicated antibodies. GAPDH was used as a loading control. The values of the band intensity represent the densitometric estimation of each band normalized by GAPDH (a). Schematic illustration of mechanism for ISL effect on ES-2 cells (b).
